# Trends in Mortality Due to Stroke in South America between 1990 and 2019

**DOI:** 10.3390/epidemiologia5030040

**Published:** 2024-09-03

**Authors:** Alexandre Castelo Branco Araujo, Orivaldo Florencio de Souza, Filomena Euridice Carvalho de Alencar, Betina Bolina Kersanach, Victor Lopes Feitosa, Julia Silva Cesar Mozzer, Vinicius Andreata Brandão, Gabriel Marim Roni, Carlos Bandeira de Mello Monteiro, Luiz Carlos de Abreu

**Affiliations:** 1 Study Design and Scientific Writing Laboratory, Health Sciences Center, Federal University of Espírito Santo, Vitória CEP29043-900, Brazil; filomena.alencar@ufes.br (F.E.C.d.A.); betina.bk.boker@gmail.com (B.B.K.); victorlopesmed@gmail.com (V.L.F.); juliascmozzer@gmail.com (J.S.C.M.); viniciusandreatab@gmail.com (V.A.B.); gabrielmroni@gmail.com (G.M.R.); luizcarlos@usp.br (L.C.d.A.); 2 Postgraduate Program in Nutrition and Health, Federal University of Espírito Santo, Vitória CEP29043-900, Brazil; orivaldo.souza@ufac.br; 3 School of Arts, Sciences and Humanities, University of São Paulo, São Paulo CEP03828-000, Brazil; carlosmonteiro@usp.br; 4 Postgraduate Program in Medical Sciences, University of São Paulo, São Paulo CEP01246-903, Brazil

**Keywords:** stroke, South America, trend, mortality, joinpoint regression

## Abstract

Stroke is the second leading cause of death and disability in Latin America; however, few epidemiological studies have been conducted in South America. An observational study was conducted to analyze trends in stroke mortality in South American (SA) countries. Age-standardized mortality rates and proportional mortality due to stroke in the populations of SA countries between 1990 and 2019 were assessed by extracting data from the Global Burden of Disease 2019 study. Joinpoint regression models were employed to identify trends in the annual percentage change in mortality rates for each segment. Considering the data collected over the 30 years that were studied, the age-standardized stroke mortality trend decreased in Argentina (−1.6%), Uruguay (−0.6%), Brazil (−0.5%), Guyana (−0.5%), and Bolivia (−0.4%), while Venezuela (+1.6%) and Suriname (+1.0%) showed an increasing trend. The proportional stroke mortality trend decreased in Argentina (−1.7%), Paraguay (−0.9%), Uruguay (−0.7%), Guyana (−0.7%), Brazil (−0.5%), and Chile (−0.5%), whereas Bolivia (+1.0%), Suriname (+0.6%), and Peru (+0.4%) exhibited an increasing trend. The trends in stroke mortality between 1990 and 2019 demonstrated considerable variability. While most SA countries experienced significant decreases in stroke mortality trends, Venezuela and Suriname showed increases in age-standardized mortality rates, and Bolivia, Suriname, and Peru exhibited increases in proportional mortality rates. No decreasing stroke mortality trend was observed in the segment after the last joinpoint, highlighting the need for improvement in prevention and treatment.

## 1. Introduction

Stroke is the second most common cause of death and the leading cause of long-term neurological disability in adults worldwide [1,2,3]. In 2019, there were 12.2 million new cases, 6.55 million deaths, and 143 million disability-adjusted life years due to stroke globally, highlighting its significant social and economic impacts [4,5].

Global data on the magnitude of health loss due to diseases analyzed in the Global Burden of Disease (GBD) study between 1990 and 2017 revealed that, in recent decades, there has been a global shift in the impact caused by the reduction in communicable diseases with respect to non-communicable diseases [6]. Among these, stroke accounted for around 11% of all deaths worldwide in 2019 [3]. This is especially pronounced in low- and lower-middle-income countries, where mortality rates are 3.6 times higher, and 86% of recorded deaths are attributable to stroke [4,5,6,7,8].

Epidemiological studies carried out between 1975 and 2019 in the United States of America and between 1996 and 2015 in the European Union have shown a downward trend in stroke mortality rates, although there is regional variability, particularly in high-income countries [9,10,11]. There were similar findings in the Middle East and North Africa, as well as in China, where there was a significant decline in stroke mortality rates between 1990 and 2019 [11,12,13,14].

Advancements in the outpatient treatment of modifiable risk factors and improvements in pre-hospital and hospital care for acute stroke may partially explain the observed downward trends, but they depend on the degree of technological development and access to a qualified health system in each country [4,15,16,17]. Despite these advances, with the increase in population size and life expectancy, the absolute number of new cases and deaths has tended to rise, which has socioeconomic repercussions given the high numbers of hospitalizations, deaths, sequelae, and potential years of life lost [4,15,18].

Stroke mortality has been understudied in South America [1,19,20]. Research conducted in Latin American and Caribbean countries between 1979 and 2015 indicates a decreasing trend in mortality rates [21]. Nevertheless, stroke is the second leading cause of death and disability in adults in the region, with 600,000 new cases and 260,000 deaths reported in 2017, reflecting an 81% increase in incident cases and a 40% increase in the number of deaths compared to 1990 [20,21,22]. Given the public health burden, the limited research available in South America, and the high morbidity and mortality associated with stroke, along with the varying social, cultural, and economic contexts of each country, an updated assessment of the subject is necessary. Thus, the aim of this study was to assess the temporal trends in stroke mortality in South American (SA) countries from 1990 to 2019.

## 2. Materials and Methods

### 2.1. Overview and Input Data

This was an observational study that used data from the GBD 2019 study on stroke in the population of SA countries from 1990 to 2019. The GBD study is a public database (https://ghdx.healthdata.org/ (accessed on 1 April 2024)) collated by the Institute for Health Metrics and Evaluation at the University of Washington.

The GBD study provides a systematic and comprehensive assessment of 369 causes of death worldwide, covering 204 countries and territories from 1990 to 2019 [23]. For deaths, the input data were from different data sources, such as censuses, civil registrations, and vital statistics. This study was designed in accordance with the Guidelines for Accurate and Transparent Health Estimate Reporting [23,24].

### 2.2. Data Extraction

The information was extracted from the GBD study on the following website: https://vizhub.healthdata.org/gbd-results/ (accessed on 1 April 2024). The estimate used was the cause of death or injury. The epidemiological measure extracted was the number of deaths. Deaths were expressed as the rate per 100,000 inhabitants and as a percentage. The specific cause of death extracted was stroke, consisting of ischemic stroke, intracerebral hemorrhage, and subarachnoid hemorrhage [6,7,9]. Stroke has been defined according to the criteria of the World Health Organization (WHO) as “rapidly developed clinical signs of focal (or global) disturbance of cerebral function, lasting more than 24 h or leading to death, with no apparent cause other than of vascular origin” [4,15,25]. This definition excludes cases of transient ischemic attacks [15,25].

In terms of location, the SA countries selected were Argentina, Bolivia, Brazil, Chile, Colombia, Ecuador, Guyana, Paraguay, Peru, Suriname, Uruguay, and Venezuela. The locations excluded from extraction were the overseas territories of France, the United Kingdom, and the Netherlands located in South America. All information was extracted for all ages and both sexes for each year from 1990 to 2019. Age was standardized by the GBD global standard population.

### 2.3. Data Analysis

In the data analysis, first, descriptive data were visualized for the number of deaths, age-standardized mortality rate, and proportional mortality for SA countries in the years 1990 and 2019. In addition, temporal trend analyses were presented for the age-standardized mortality rate and proportional mortality. Data on the number of deaths, the mortality rate (per 100,000 inhabitants) standardized by age, and proportional mortality, with their respective 95% uncertainty intervals, were extracted into comma-separated value files. Maps of South America with subdivisions by country showed the number of deaths for the years 1990 and 2019, and they were created with the help of the Geoda spatial analysis program.

The average annual percentage change (AAPC) and annual percentage change (APC) in the age-standardized mortality rate and proportional mortality of SA countries were estimated using the Joinpoint Regression Program (version 5.0.2, 2023). Joinpoint regression models were used to identify change points in the time series and the trend of each segment [26]. The dependent variable in each model was the age-standardized mortality rate or proportional mortality for each country in South America. The independent variable in all models was the year in the period from 1990 to 2019. The model analysis options were the following: constant variation, a maximum number of five joinpoints, and logarithmic transformation. The weighted Bayesian information criterion method was applied to select the best models.

The AAPC and the APC with 95% confidence intervals estimated using the parametric method indicated the direction and magnitude of temporal trends. Joinpoint regression models with a *p*-value of <0.05 were those in which the hypothesis of an annual increase in positive values or a decrease in negative values was accepted. *p*-values of >0.05 indicated that the hypothesis of stability in the temporal trend was accepted.

### 2.4. Legal and Ethical Aspects

Due to the study design and considering that all data used are publicly available, ethical approval was not required.

## 3. Results

Over the 30 years, considering the twelve countries in South America, the absolute number of deaths due to stroke, at all ages and for both sexes, increased from 184,251 in 1990 to 228,661 in 2019 (24.1%). In the comparative analysis for each country, considering the years 1990 and 2019, it was observed that the largest increases occurred in Venezuela (130.5%) and Suriname (92.8%), followed by Ecuador (75.4%), Bolivia (66.4%), Paraguay (56.4%), Colombia (43.6%), Chile (31.1%), Brazil (24.0%), and Peru (17.1%). Three countries showed a decrease, with reductions of 14.6% in Argentina, 15.0% in Guyana, and 9.6% in Uruguay (Table 1).

The age-standardized stroke mortality rates per 100,000 inhabitants of both sexes are shown in Table 2. Comparing 1990 and 2019, there was variation in the mortality rates, with decreases in Argentina, Peru, Uruguay, Guyana, Brazil, Bolivia, Paraguay, Chile, and Colombia. In Ecuador, the mortality rate remained the same, while Venezuela and Suriname showed increases. Argentina, Brazil, Chile, Guyana, and Uruguay had the highest mortality rates, and despite the reduction, they remained at high levels.

The average annual percentage change in the age-standardized stroke mortality rate per 100,000 inhabitants between 1990 and 2019 demonstrated a decreasing mortality trend of 1.6% in Argentina (*p* < 0.001), 0.6% in Uruguay (*p* = 0.010), 0.5% in Brazil (*p* < 0.001), 0.5% in Guyana (*p* = 0.011), and 0.4% in Bolivia (*p* < 0.001). Chile (*p* = 0.466), Colombia (*p* = 0.662), Ecuador (*p* = 0.970), Paraguay (*p* = 0.658), and Peru (*p* = 0.058) showed stationary trends, while Venezuela and Suriname showed increasing trends of 1.6% (*p* < 0.001) and 1.0% (*p* = 0.007), respectively (Table 2).

Significant variability was identified for proportional mortality in South America. Stroke was responsible for 5.1% to 15.1% of total deaths in each country in 1990 and 5.8% to 13.1% in 2019. Guyana, Uruguay, Paraguay, Argentina, Brazil, Chile, Colombia, and Ecuador showed a reduction in proportional mortality when comparing the years 1990 and 2019, while Bolivia, Peru, and Venezuela displayed an increase (Table 3). The temporal trend of proportional mortality through the AAPC between 1990 and 2019 revealed a decreasing trend of 1.7% in Argentina (*p* < 0.001), 0.9% in Paraguay (*p* < 0.001), 0.7% in Uruguay (*p* < 0.001), 0.7% in Guyana (*p* < 0.001), 0.5% in Brazil (*p* < 0.001), and 0.5% in Chile (*p* < 0.001). Colombia (*p* = 0.938), Ecuador (*p* = 0.743), and Venezuela (*p* = 0.283) showed stationary trends, while Bolivia (*p* < 0.001), Suriname (*p* < 0.001), and Peru (*p* = 0.036) displayed increasing trends of 1.0%, 0.6%, and 0.4%, respectively (Table 3).

When evaluating the APC in the age-standardized stroke mortality rate between 1990 and 2019 using the joinpoint regression model, several points of change in the mortality trend were identified throughout the time series in different countries. In the segment determined after the last joinpoint, the mortality trend increased by 7.46% in Venezuela after 2014 (*p* < 0.001), 3.24% in Suriname after 2011 (*p* < 0.001), 2.31% in Colombia after 2013 (*p* < 0.001), 2.11% in Chile after 2016 (*p* = 0.005), 1.18% in Brazil after 2014 (*p* = 0.001), 1.17% in Ecuador after 2010 (*p* = 0.001), 1.14% in Argentina after 2013 (*p* = 0.002), and 1.01% in Guyana after 2000 (*p* < 0.001). The trend was stationary in Bolivia after 2014 (*p* = 0.582), Paraguay after 2015 (*p* = 0.089), Peru after 2016 (*p* = 0.296), and Uruguay after 2014 (*p* = 0.241) (Table 4).

In the same way, in the analysis of the APC in proportional mortality, several points of change in the trend throughout the time series were also identified. In the segment determined after the last joinpoint, there was an increasing proportional mortality trend of 1.11% in Brazil after 2017 (*p* = 0.008), 1.02% in Suriname after 2012 (*p* < 0.001), 0.81% in Ecuador after 2014 (*p* = 0.015), 0.78% in Paraguay after 2015 (*p* = 0.039), 0.71% in Colombia after 2013 (*p* = 0.001), 0.45% in Bolivia after 2014 (*p* < 0.001), 0.43% in Argentina after 2012 (*p* < 0.001), 0.39% in Guyana after 2000 (*p* < 0.001), and 0.30% in Peru after 2002 (*p* = 0.019). The trend was stationary in Chile after 2015 (*p* = 0.998), Uruguay after 2014 (*p* = 0.369), and Venezuela after 2014 (*p* = 0.09) (Table 5).

## 4. Discussion

Throughout the time series, the age-standardized stroke mortality trend in SA countries demonstrated considerable variability, decreasing by 1.6% in Argentina, 0.6% in Uruguay, 0.5% in Brazil, 0.5% in Guyana, and 0.4% in Bolivia. Chile, Colombia, Ecuador, Paraguay, and Peru showed a stationary trend, while Venezuela and Suriname showed increasing trends of 1.6% and 1.0%, respectively.

In the analysis using the joinpoint regression model, several points of change in the age-standardized mortality trend were observed between 1990 and 2019. In the time segment after the last joinpoint, an increasing trend was detected in Venezuela, Suriname, Argentina, Colombia, Chile, Brazil, Ecuador, and Guyana, and a stationary trend was detected in Bolivia, Paraguay, Peru, and Uruguay. No SA country had a decreasing age-standardized mortality trend in the last time segment.

The proportional mortality trend due to stroke between 1990 and 2019 decreased by 1.7% in Argentina, 0.9% in Paraguay, 0.7% in Guyana, 0.7% in Uruguay, 0.5% in Brazil, and 0.5% in Chile. Colombia, Ecuador, and Venezuela showed stationary trends, while Bolivia (1%), Suriname (0.6%), and Peru (0.4%) showed increasing trends. Again, in the analysis of the time segment after the last joinpoint, an increasing or stationary trend was observed for all SA countries, revealing the importance of continuous surveillance and the need to intensify prevention and treatment programs for stroke in South America.

Comparing the years 1990 and 2019, the present study detected an increase of 24.1% in the absolute number of deaths due to stroke in South America, with Venezuela, Suriname, Ecuador, Bolivia, Paraguay, and Colombia showing the largest increases, followed by Chile, Brazil, and Peru. A decrease in the absolute number of deaths was only evident in Guyana, Argentina, and Uruguay.

Analyzing the high number and absolute increase in deaths due to stroke observed in most SA countries between 1990 and 2019, it was concluded that, despite advances in diagnosis and treatment in recent years, stroke remains a public health problem, with social and economic impacts being evident through the hospitalizations, deaths, and disabilities. This corroborates the findings of Soto et al. in their study in Latin American and Caribbean countries between 1979 and 2015 [21]. On the other hand, Guyana, Argentina, and Uruguay showed a reduction in the absolute number of deaths, as observed in the USA [9] and in European Union countries [10].

Substantial variability in age-standardized stroke mortality rates per 100,000 inhabitants occurred in 2019 in SA countries, with the lowest rates in Peru (25.9), Ecuador (34.6), and Colombia (35.4) and the highest rates in Guyana (108.6), Uruguay (102.5), and Suriname (99.5). The reduction in age-standardized mortality rates between 1990 and 2019 identified in Argentina, Peru, Uruguay, Guyana, Brazil, Bolivia, Paraguay, Chile, and Colombia has been found in other countries and regions of the world [9,10,12,13]. Despite this, the mortality rates for most countries in SA were higher than those found in the USA in 2019 (30.9 for women and 38.7 for men) and in the European Union in 2015 (41.99 for both sexes) [9,10], and they were closer to the values identified in the Middle East and North Africa in 2019 (87.7 for both sexes) [12].

These findings suggest that an unhealthy lifestyle, limited access to outpatient treatment of the main modifiable risk factors (hypertension, obesity, diabetes mellitus, dyslipidemia), less early recognition of an acute episode of stroke, the difficulties in quality hospital care for reperfusion therapy (venous thrombolysis or endovascular treatment for ischemic stroke), and the lack of rehabilitation services may lead to higher mortality in middle-income countries, such as South America, in comparison with high-income countries [1,4,11,21,27].

The temporal trend analyses of age-standardized stroke mortality carried out by Ananth et al. in the USA between 1975 and 2019 [9], by Soto et al. in the European Union between 1996 and 2015 [10], and by Soto et al. in Latin America and the Caribbean between 1979 and 2015 [21] showed downward trends in stroke mortality rates of 2.7%, 4.2%, and 1.9%, respectively, but with variations among the countries analyzed. Similar results were also observed in South America, with decreasing age-standardized stroke mortality trends in Argentina, Uruguay, Brazil, Guyana, and Bolivia. Nevertheless, other SA countries demonstrated a stationary trend, while Venezuela and Suriname demonstrated increasing trends. These aspects confirm the variability in stroke mortality trends in South America and reinforce the importance of advancing health policies and technological development focused mainly on countries with higher mortality.

Wang et al., researching stroke incidence and mortality worldwide, observed a global 36.4% decrease in age-standardized stroke mortality rates per 100,000 inhabitants when comparing the years 1990 and 2019, with greater drops for high-income countries in Asia, North America, Latin America, Western Europe, and Australia [11]. In South America, there was a reduction in age-standardized stroke mortality rates in many countries, despite the fact that some countries continued to experience high mortality rates and increasing trends, indicating that the burden of stroke in the region still requires greater investment in prevention, hospital care, and rehabilitation [11,20,21].

In addition, it was noted that after 2014, there was a slowdown in the decline in stroke mortality, as it became practically unchanged in some regions of the world [28]. When evaluating the stroke mortality trends in South America, it was observed that no country showed a decreasing trend in mortality rates in the time segment after the last joinpoint. The trend increased in Argentina after 2013, Brazil after 2014, Chile after 2016, Colombia after 2013, Ecuador after 2010, Guyana after 2000, Suriname after 2011, and Venezuela after 2014, and it was stationary in Bolivia after 2014, Paraguay after 2015, Peru after 2016, and Uruguay after 2014. Monitoring these trends is extremely important due to the possibility of an increase in the number of deaths in the coming years and the need to outline new preventive and acute care measures through the dissemination of specialized stroke units and better access to cerebral reperfusion techniques for ischemic stroke.

According to WHO data, in 2019, stroke was responsible for around 11% of the total deaths worldwide [3]. In the present study, it was observed that in 1990, proportional mortality due to stroke presented percentages close to or above these values in Guyana, Uruguay, Paraguay, Argentina, Suriname, Brazil, and Chile, while Bolivia, Colombia, Ecuador, Peru, and Venezuela presented lower proportional mortality rates. When compared with 2019, a reduction was observed for most SA countries; however, the levels were still high for Suriname (13.1%), Guyana (12.5%), and Uruguay (10.4%), revealing that the burden caused by stroke is still important in some SA countries.

Stroke is a multifactorial disease, and according to global data, approximately 90.5% of cases are preventable [16]. Modifiable risk factors include behavioral factors (smoking, inadequate diet, alcohol intake, and low physical activity), metabolic factors (high body mass index, diabetes mellitus, hypertension, high total cholesterol), and environmental factors (air pollution and lead exposure) [1,16,17,29]. A study carried out in Latin America and the Caribbean identified metabolic risk factors as the principal contributors—particularly, high systolic blood pressure and high body mass index [30]. Although this study did not investigate risk factors in South America, it is crucial to conduct research in this area to better understand and develop public policies for primary prevention targeting the most prevalent risk factors in South America.

This study has some limitations. Firstly, the lack of high-quality primary data from each country in South America and the need to use alternative data methods could have compromised the analysis and led to errors in the estimates. Secondly, as this was an ecological study using an international database while considering the population group as a whole, caution must be taken when transposing the results to the individual level or specific groupings. Thirdly, some regions of South America are impoverished and difficult to access, which can significantly interfere with diagnosis and, consequently, affect stroke incidence and mortality. This underscores the need for local studies on the effective allocation of financial resources and the development of control measures based on concrete, locally sourced data. In addition, the analysis considered both ischemic and hemorrhagic stroke simultaneously even though the risk factors and treatments used are different, which would justify future individualized studies to organize specialized teams for the different types of strokes to ensure rapid recognition and treatment.

## 5. Conclusions

Temporal trends in age-standardized mortality rates and proportional mortality due to stroke for both sexes between 1990 and 2019 showed considerable variability among SA countries. The age-standardized stroke mortality trend decreased in Argentina, Uruguay, Brazil, Guyana, and Bolivia; was stationary in Chile, Colombia, Ecuador, Paraguay, and Peru; and increased in Venezuela and Suriname. The proportional stroke mortality trend decreased or was stationary in most SA countries, except in Bolivia, Suriname, and Peru, which showed increasing trends. In the analysis of the segment after the last joinpoint, it was observed that no countries in South America manifested a decreasing stroke mortality trend. The absolute number of deaths rose over the 30-year time series in SA countries, except in Argentina, Guyana, and Uruguay, which demonstrated a decline.

Given these findings, it is clear that stroke is a public health problem in SA countries despite advances in the recognition of risk factors, the availability of imaging exams for diagnosis, and access to international treatment protocols. Considering the fact that stroke is a highly preventable disease, this reinforces the importance of effective preventive measures, outpatient treatment of cardiovascular risk factors, and adequate hospital care for those affected to minimize deaths and disabilities. Future research on the predominant risk factors, approaches to acute patients, and rehabilitation services in South America is essential for improving stroke epidemiological outcomes. Additionally, implementing monitoring systems will be critical for ensuring the effectiveness of strategies and adjusting interventions.

## Figures and Tables

**Table 1:** Number of deaths due to stroke in South America in 1990 and 2019.

	1990	2019
	Number of Deaths (UI 95% *)	Number of Deaths (UI 95% *)
Argentina	30,540.4 (28,897.1; 31,697.3)	26,065.9 (23,536.7; 28,162.5)
Bolivia	3177.9 (2513.0; 3870.5)	5288.7 (3866.2; 6991.9)
Brazil	105,603.9 (100,300.3; 109,634.9)	131,007.0 (119,134.6; 139,017.7)
Chile	8044.0 (7623.8; 8403.6)	10,545.6 (9280.2; 11,476.7)
Colombia	11,793.2 (11,027.0; 12,510.4)	6943.2 (13,052.9; 21,269.5)
Ecuador	3472.7 (3122.8; 3678.8)	6091.4 (4924.1; 7631.2)
Guyana	985.7 (866.1; 1100.6)	837.2 (663.1; 1047.9)
Paraguay	2126.6 (1863.8; 2355.1)	3326.9 (2538.5; 4341.1)
Peru	7538.2 (6542.7; 8639.6)	8833.4 (6556.8; 11,906.4)
Suriname	297.3 (272.4; 316.3)	73.4 (482.8; 674.6)
Uruguay	3897.7 (3632.6; 4103.1)	3522.8 (3073.4; 3864.6)
Venezuela	6779.4 (6345.0; 7188.8)	15,631.5 (12,268.3; 19,933.1)

* UI 95%: 95% uncertainty interval.

**Table 2:** Age-standardized stroke mortality rate and average annual percentage change in South America between 1990 and 2019.

	1990	2019	Average Annual Percentage Change (CI 95% **)	*p*	Interpretation
	Mortality Rate * (UI 95%)	Mortality Rate * (UI 95%)			
Argentina	92.2 (87.2; 95.7)	57.7 (52.1; 62.4)	−1.6 (−2.1; −1.2)	<0.001	Decreasing
Bolivia	49.4 (39.1; 60.2)	44.0 (32.1; 58.2)	−0.4 (−0.5; −0.3)	<0.001	Decreasing
Brazil	70.9 (67.3; 73.6)	60.4 (54.9; 64.1)	−0.5 (−0.7; −0.3)	<0.001	Decreasing
Chile	60.5 (57.4; 63.2)	57.9 (50.9; 63.0)	−0.1 (−0.5; 0.2)	0.466	Stationary
Colombia	36.2 (33.8; 38.4)	35.4 (27.3; 44.5)	−0.1 (−0.8; 0.5)	0.662	Stationary
Ecuador	34.6 (31.1; 36.6)	34.6 (28.0; 43.3)	0.0 (−0.6; 0.6)	0.970	Stationary
Guyana	128.0 (112.4; 142.9)	108.6 (86.0; 135.9)	−0.5 (−0.9; −0.1)	0.011	Decreasing
Paraguay	52.5 (46.0; 58.2)	48.0 (36.6; 62.6)	−0.2 (−0.9; 0.6)	0.658	Stationary
Peru	34.6 (30.1; 39.7)	25.9 (19.2; 35.0)	−0.9 (−1.8; 0.0)	0.058	Stationary
Suriname	76.9 (70.4; 81.8)	99.5 (83.8; 117.1)	1.0 (0.3; 1.7)	0.007	Increasing
Uruguay	124.1 (115.7; 130.7)	102.5 (89.4; 112.4)	−0.6 (−1.0; −0.1)	0.010	Decreasing
Venezuela	36.0 (33.7; 38.1)	55.6 (43.7; 71.0)	1.6 (0.9; 2.3)	<0.001	Increasing

* Age-standardized mortality rate per 100,000 inhabitants; UI 95%: 95% uncertainty interval; ** CI 95%: 95% confidence interval.

**Table 3:** Proportional stroke mortality and annual percentage change in South America between 1990 and 2019.

	1990	2019	Average Annual Percentage Change (CI 95% **)	*p*	Interpretation
	Proportional Mortality (UI 95% *)	Proportional Mortality (UI 95% *)			
Argentina	12.1 (11.4; 12.5)	7.4 (6.7; 8.1)	−1.7 (−1.8; −1.5)	<0.001	Decreasing
Bolivia	5.1 (4.1; 6.1)	6.9 (5.4; 8.4)	1.0 (0.9; 1.1)	<0.001	Increasing
Brazil	10.7 (10.1; 11.1)	9.2 (8.4; 9.8)	−0.5 (−0.6; −0.4)	<0.001	Decreasing
Chile	10.6 (10.0; 11.0)	9.3 (8.2; 10.0)	−0.5 (−0.6; −0.3)	<0.001	Decreasing
Colombia	6.9 (6.4: 7.3)	6.8 (5.9; 7.6)	0.0 (−0.2; 0.2)	0.938	Stationary
Ecuador	6.7 (5.9; 7.1)	6.5 (6.0; 7.1)	−0.1 (−0.5; 0.4)	0.743	Stationary
Guyana	15.1 (14.4; 15.8)	12.5 (11.5; 13.5)	−0.7 (−1.0; −0.4)	<0.001	Decreasing
Paraguay	12.7 (11.3; 13.5)	9.7 (8.4; 12.0)	−0.9 (−1.0; −0.7)	<0.001	Decreasing
Peru	5.3 (4.8; 5.8)	5.8 (4.9; 6.9)	0.4 (0.0; 0.8)	0.036	Increasing
Suriname	11.0 (10.1; 11.7)	13.1 (11.8; 14.2)	0.6 (0.5; 0.8)	<0.001	Increasing
Uruguay	13.1 (12.3; 13.8)	10.4 (9.1; 11.3)	−0.7 (−0.9; −0.6)	<0.001	Decreasing
Venezuela	7.6 (7.1; 8.0)	8.3 (7.4; 9.1)	0.3 (−0.3; 0.9)	0.283	Stationary

* UI 95%: 95% uncertainty interval; ** CI 95%: 95% confidence interval.

**Table 4:** APC in the age-standardized stroke mortality rate (100,000 inhabitants) in South America between 1990 and 2019.

	Segment	Annual Percentage Change (CI 95% *)	*p*	Interpretation
Argentina	1990–2003	−2.2 (−2.4; −2.0)	<0.001	Decreasing
	2003–2008	−3.5 (−4.7; −2.3)	<0.001	Decreasing
	2008–2013	−1.1 (−2.3; 0.2)	0.088	Stationary
	2013–2019	1.1 (0.5; 1.8)	0.002	Increasing
Bolivia	1990–1993	−0.7 (−1.1; −0.2)	0.004	Decreasing
	1993–2003	−1.7 (−1.8; −1.6)	<0.001	Decreasing
	2003–2006	−0.1 (−1.0; 0.8)	0.779	Stationary
	2006–2014	0.9 (0.8; 1.1)	<0.001	Increasing
	2014–2019	0.1 (−0.1; 0.2)	0.582	Stationary
Brazil	1990–2001	−1.3 (−1.5; −1.1)	<0.001	Decreasing
	2001–2014	−0.6 (−0.8; −0.4)	<0.001	Decreasing
	2014–2019	1.2 (0.5; 1.8)	0.001	Increasing
Chile	1990–1992	−4.1 (−6.7; −1.5)	0.005	Decreasing
	1992–1997	−1.1 (−1.9; −0.2)	0.017	Decreasing
	1997–2008	0.0 (−0.2; 0.2)	0.953	Stationary
	2008–2011	2.2 (−0.4; 5.0)	0.095	Stationary
	2011–2016	−0.6 (−1.5; 0.2)	0.130	Stationary
	2016–2019	2.1 (0.8; 3.5)	0.005	Increasing
Colombia	1990–2013	−0.9 (−1.0; −0.7)	<0.001	Decreasing
	2013–2019	2.3 (1.2; 3.4)	<0.001	Increasing
Ecuador	1990–2001	−2.1 (−2.5; −1.6)	<0.001	Decreasing
	2001–2004	5.5 (−1.3; 12.8)	0.111	Stationary
	2004–2010	−1.1 (−2.5; 0.4)	0.148	Stationary
	2010–2019	1.2 (0.6; 1.8)	0.001	Increasing
Guyana	1990–1996	−2.1 (−2.9; −1.2)	<0.001	Decreasing
	1996–2000	−4.7 (−7.1; −2.2)	0.001	Decreasing
	2000–2019	1.0 (0.9; 1.2)	<0.001	Increasing
Paraguay	1990–1995	1.3 (−0.1; 2.7)	0.072	Stationary
	1995–2002	−2.5 (−3.5; −1.4)	<0.001	Decreasing
	2002–2005	3.2 (−3.1; 9.8)	0.304	Stationary
	2005–2015	−1.0 (−1.5; −0.4)	0.002	Decreasing
	2015–2019	1.7 (−0.3; 3.7)	0.089	Stationary
Peru	1990–1995	0.1 (−1.4; 1.6)	0.884	Stationary
	1995–2001	−4.2 (−5.6; −2.7)	<0.001	Decreasing
	2001–2007	−2.0 (−3.5; −0.5)	0.012	Decreasing
	2007–2010	6.8 (−0.3; 14.4)	0.058	Stationary
	2010–2016	−2.1 (−3.6; −0.6)	0.010	Decreasing
	2016–2019	1.7 (−1.7; 5.3)	0.296	Stationary
Suriname	1990–1993	4.9 (2.5; 7.4)	<0.001	Increasing
	1993–1996	−7.6 (−11.8; −3.1)	0.003	Decreasing
	1996–2002	5.9 (4.8; 7.1)	<0.001	Increasing
	2002–2011	−2.5 (−3.0; −2.0)	<0.001	Decreasing
	2011–2019	3.2 (2.7; 3.8)	<0.001	Increasing
Uruguay	1990–2003	0.6 (0.3; 1.0)	<0.001	Increasing
	2003–2014	−2.6 (−3.1; −2.2)	<0.001	Decreasing
	2014–2019	0.8 (−0.6; 2.2)	0.241	Stationary
Venezuela	1990–2014	0.4 (0.2; 0.5)	<0.001	Increasing
	2014–2019	7.5 (5.6; 9.4)	<0.001	Increasing

* CI 95%: 95% confidence interval.

**Table 5:** APC in proportional stroke mortality in South America between 1990 and 2019.

	Segment	Annual Percentage Change (CI 95% *)	*p*	Interpretation
Argentina	1990–1993	−1.3 (−1.9; −0.8)	<0.001	Decreasing
	1993–2000	−2.8 (−3.0; −2.7)	<0.001	Decreasing
	2000–2003	−1.7 (−2.8; −0.6)	0.005	Decreasing
	2003–2008	−2.9 (−3.2; −2.6)	<0.001	Decreasing
	2008–2012	−1.8 (−2.3; −1.2)	<0.001	Decreasing
	2012–2019	0.4 (0.3; 0.6)	<0.001	Increasing
Bolivia	1990–1994	2.0 (1.7; 2.3)	<0.001	Increasing
	1994–1997	1.3 (0.5; 2.2)	0.005	Increasing
	1997–2002	0.7 (0.5; 1.0)	<0.001	Increasing
	2002–2014	1.0 (1.0; 1.1)	<0.001	Increasing
	2014–2019	0.5 (0.3; 0.6)	<0.001	Increasing
Brazil	1990–1993	0.7 (0.3; 1.0)	0.003	Increasing
	1993–1996	−1.0 (−1.8; −0.3)	0.013	Decreasing
	1996–2007	−0.7 (−0.8; −0.7)	<0.001	Decreasing
	2007–2014	−1.1 (−1.2; −0.9)	<0.001	Decreasing
	2014–2017	0.1 (−0.7; 0.8)	0.849	Stationary
	2017–2019	1.1 (0.3; 1.9)	0.008	Increasing
Chile	1990–1996	−1.3 (−1.4; −1.1)	<0.001	Decreasing
	1996–2003	0.0 (−0.1; 0.2)	0.585	Stationary
	2003–2006	−0.5 (−1.6; 0.5)	0.302	Stationary
	2006–2012	0.1 (−0.1: 0.3)	0.353	Stationary
	2012–2015	−1.6 (−2.7; −0.6)	0.006	Decreasing
	2015–2019	0.0 (−0.3; 0.3)	0.998	Stationary
Colombia	1990–1996	0.5 (0.2; 0.9)	0.008	Increasing
	1996–2001	−1.1 (−1.8; −0.4)	0.003	Decreasing
	2001–2005	1.2 (0.1; 2.3)	0.029	Increasing
	2005–2013	−0.9 (−1.2; −0.6)	<0.001	Decreasing
	2013–2019	0.7 (0.3; 1.1)	0.001	Increasing
Ecuador	1990–1992	1.7 (−1.1; 4.6)	0.213	Stationary
	1992–2000	−1.9 (−2.2; −1.5)	<0.001	Decreasing
	2000–2003	1.3 (−1.4; 4.2)	0.320	Stationary
	2003–2011	−0.9 (−1.3; −0.6)	<0.001	Decreasing
	2011–2014	3.0 (0.2; 5.9)	0.038	Increasing
	2014–2019	0.8 (0.2; 1.4)	0.015	Increasing
Guyana	1990–1996	−1.9 (−2.5; −1.3)	<0.001	Decreasing
	1996–2000	−3.9 (−5.6; −2.3)	<0.001	Decreasing
	2000–2019	0.4 (0.3; 0.5)	<0.001	Increasing
Paraguay	1990–1995	0.2 (−0.3; 0.7)	0.444	Stationary
	1995–2002	−2.3 (−2.7; −1.9)	<0.001	Decreasing
	2002–2007	0.3 (−0.4; 1.1)	0.379	Stationary
	2007–2015	−1.9 (−2.2; −1.6)	<0.001	Decreasing
	2015–2019	0.8 (0.0; 1.5)	0.039	Increasing
Peru	1990–1995	3.2 (1.6; 4.8)	<0.001	Increasing
	1995–2002	−1.2 (−2.4; 0.0)	0.044	Decreasing
	2002–2019	0.3 (0.1; 0.5)	0.019	Increasing
Suriname	1990–1993	2.4 (2.0; 2.9)	<0.001	Increasing
	1993–1996	−0.9 (−1.8; −0.1)	0.036	Decreasing
	1996–1999	4.0 (3.1; 4.9)	<0.001	Increasing
	1999–2004	1.7 (1.4; 2.0)	<0.001	Increasing
	2004–2012	−1.7 (−1.8; −1.6)	<0.001	Decreasing
	2012–2019	1.0 (0.9; 1.1)	<0.001	Increasing
Uruguay	1990–2004	0.4 (0.3; 0.6)	<0.001	Increasing
	2004–2014	−2.9 (−3.1; −2.6)	<0.001	Decreasing
	2014–2019	0.3 (−0.4; 1.0)	0.369	Stationary
Venezuela	1990–2014	−0.2 (−0.5; 0.1)	0.200	Stationary
	2014–2019	2.7 (−0.4; 5.9)	0.090	Stationary

* CI 95%: 95% confidence interval.

## Data Availability

Data were extracted from: https://vizhub.healthdata.org/gbd-results/ (accessed on 1 April 2024).
